# Intrinsic Photosensitive Retinal Ganglion Cells in the Diurnal Rodent, *Arvicanthis* *ansorgei*

**DOI:** 10.1371/journal.pone.0073343

**Published:** 2013-08-09

**Authors:** Diana Karnas, David Hicks, Jérôme Mordel, Paul Pévet, Hilmar Meissl

**Affiliations:** 1 Neuroanatomical Department, Max Planck Institute for Brain Research, Frankfurt/M, Germany; 2 Institute for Cellular and Integrative Neuroscience, CNRS UPR-3212 Strasbourg University, Strasbourg, France; Karlsruhe Institute of Technology, Germany

## Abstract

Intrinsically photosensitive retinal ganglion cells (ipRGCs) represent a new class of photoreceptors which support a variety of non-image forming physiological functions, such as circadian photoentrainment, pupillary light reflex and masking responses to light. In view of the recently proposed role of retinal inputs for the regulation of diurnal and nocturnal behavior, we performed the first deep analysis of the ipRGC system in a diurnal rodent model, *Arvicanthis* *ansorgei* , and compared the anatomical and physiological properties of ipRGCs with those of nocturnal mice. Based on somata location, stratification pattern and melanopsin expression, we identified two main ipRGC types in the retina of *Arvicanthis* : M1, constituting 74% of all ipRGCs and non-M1 (consisting mainly of the M2 type) constituting the following 25%. The displaced ipRGCs were rarely encountered. Phenotypical staining patterns of ganglion cell markers showed a preferential expression of Brn3 and neurofilaments in non-M1 ipRGCs. In general, the anatomical properties and molecular phenotyping of ipRGCs in *Arvicanthis* resemble ipRGCs of the mouse retina, however the percentage of M1 cells is considerably higher in the diurnal animal. Multi-electrode array recordings (MEA) identified in newborn retinas of *Arvicanthis* three response types of ipRGCs (type I, II and III) which are distinguished by their light sensitivity, response strength, latency and duration. Type I ipRGCs exhibited a high sensitivity to short light flashes and showed, contrary to mouse type I ipRGCs, robust light responses to 10 ms flashes. The morphological, molecular and physiological analysis reveals very few differences between mouse and *Arvicanthis* ipRGCs. These data imply that the influence of retinal inputs in defining the temporal niche could be related to a stronger cone input into ipRGCs in the cone-rich *Arvicanthis* retina, and to the higher sensitivity of type I ipRGCs and elevated proportion of M1 cells.

## Introduction

In mammals, non-visual photoreception provides the most important time cue for synchronization of the master clock in the suprachiasmatic nuclei (SCN) with the solar day. The time-keeping mechanism governs the periodic changes in physiology, metabolism and behavior of the organism. Interestingly, the fundamental properties of rhythmic expression of core clock genes [1], the rhythms in clock-controlled genes as part of the output pathways of the SCN [2,3], as well as the daily rhythms of SCN electrical activity [4], and the circadian response to acute light exposure [5] appear to activate the same molecular and intercellular pathways in nocturnal and diurnal animals [1,6] suggesting that the phase difference is dependent on the coupling of the circadian clock to output mechanisms [7].

Non-visual photoreception is governed by melanopsin-expressing, intrinsically photosensitive retinal ganglion cells (ipRGCs) encoding irradiance information used for photoentrainment [8,9]. Rod and cone signals, which contribute to the light response in the SCN and to circadian entrainment [10], act through activation of the ipRGCs [11,12]. In double-knockout mice lacking the photopigment melanopsin and RPE65, a key protein used in retinal chromophore recycling, which retain only a small rod input, the phase of circadian activity rhythms with respect to the external light/dark cycle is reversed from nocturnal to diurnal entrainment [13]. A similar switch from nocturnal to diurnal activity was also observed in wild-type mice under light/dark cycles with light intensities reduced to scotopic levels, suggesting that the state of photoreceptors can play an important role in determining phasing of activity in diurnal and nocturnal species [13,14]. These data imply a greater role of retinal inputs in temporal niche switching mechanisms than previously assumed, and a model was proposed consisting of two processes, a change in clock-controlled outputs and a change in the direct response to light (masking) [14].

Typical representatives of animals with predominantly diurnal or crepuscular locomotor activity rhythms are the murid rodents of the *Arvicanthis* genus [15–17]. The number of cones in the retina of *Arvicanthis* is considerably higher (~30%) than in mice or rats (2–3%) [18], and recordings of the electroretinogram (ERG) reveal typical features for diurnal mammals showing a strong contribution of cone-driven responses [19]. The mechanisms that could be responsible for determining diurnality in *Arvicanthis* are yet unknown. Therefore, given the possible role of retinal mechanisms in temporal niche switching, we characterized the non-visual system in the *Arvicanthis* retina and compared it to the nocturnal mouse.

## Materials and Methods

### Animals and Ethics Statement

Sudanian grass rats ( *Arvicanthis* *ansorgei* ) were obtained from a breeding colony of the Chronobiotron, UMS n° 3415, CNRS and University of Strasbourg, France. The C57BL/6 mice originate from the animal facility of the Max Planck Institute for Brain Research, Frankfurt, Germany. Animals were maintained under a 12 h light/ 12 h dark cycle with food and water available *ad libitum*. All studies were consistent with the ARVO Statement for the use of Animals in Ophthalmic and Vision Research, with French national laws and with the law for animal experiments issued by the German Government (Tierschutzgesetz). Killing of mice and *Arvicanthis* for organ harvesting (retinal wholemounts) were approved by the Animal Care and Use Committee of the Max Planck Institute for Brain Research, Frankfurt, and the local animal welfare officer of the respective facility and reported to the local authorities (Regierungspräsidium Darmstadt).

### Tissue preparation

Isolated retinas from male and female animals were used for both neuroanatomical and electrophysiological studies. Depending on the age, animals were either rapidly decapitated and the eyes enucleated (P0 – P7, P = postnatal day), or first deeply anaesthetized with isoflurane (DeltaSelect, Germany), then decapitated and the eyes removed (P8 – adult). After removal of the cornea, iris, sclera, pigment epithelium and vitreous the retinas were placed into 0.1 M aerated phosphate buffer (PB), pH 7.4, before they were further processed for histology. The expression of RGC (retinal ganglion cell) markers was investigated in 19 retinas from animals of age 1 to 26 months. Cell density measurements were performed in 34 retinas from day of birth (P0) to 26 months old. For electrophysiological experiments, retinas were transferred into Ames medium (Sigma-Aldrich, München, Germany) gassed with 95% O_2_ / 5% CO_2_. In these physiological experiments, four retinas of *Arvicanthis* (age: P0 – P3) and three retinas of C57BL/6 mice (age: P0 – P2) were studied.

### Immunohistochemistry

Retinas were immersed in 4% (w/v) paraformaldehyde (in 0.1 M PB, pH 7.4), for 15 min to 1 h. After fixation, retinas were cryoprotected in graded sucrose solutions (10, 20 and 30% w/v in 0.1 M PB, respectively), and stored at -20 °C until further processing. Retina wholemounts were thawed and snap frozen (3 x) on liquid nitrogen-cooled metal blocks. Tissues were pre-incubated in 0.1 M PB containing 10% normal donkey serum (NDS), 1% bovine serum albumin (BSA) and 0.5% Triton X-100 (buffer A), for 1–2 h at room temperature (RT). The primary antibody diluted in buffer A (but only 3% NDS and 0.1% NaN_3_) was incubated overnight at RT or for up to 5 days at 4 °C. Afterwards, retinas were washed with 0.1 M PB before they were incubated with the secondary antibody diluted in buffer A for 2–4 h at RT. Retinas were again washed, mounted on microscope slides and coverslipped with Aqua-Polymount medium (Polysciences, Warrington, USA). Immunohistochemistry was performed using the indirect fluorescence method. Tissues were incubated in a mixture of primary antibodies, followed by a mixture of secondary antibodies. In control experiments one or both primary antibodies were omitted.

### Antibodies

The characteristics of primary antibodies used in the present study were extensively described in a previous study [20]. For the detection of **melanopsin** we used a rabbit polyclonal antibody PA1-781 (1:1000; Affinity Bioreagents) that was shown to cross-react with rat and mouse melanopsin (manufacturer’s information [9,21]). The polyclonal **goat anti-Brn3 antibody** used in the present study (1:200; sc-6026, clone C-13; Santa Cruz Biotechnology) specifically recognizes three bands in Western blot corresponding to the three Brn3 family members which are all expressed by RGCs [22]: Brn3a at 53 kDa, Brn3b at 51 kDa and Brn3c at 42 kDa (manufacturer’s information). **Islet 1 (Isl1)** protein was shown to be expressed by RGCs, starburst amacrine cells and bipolar cells [23,24]. We used the monoclonal mouse antibody 39.4D5 (1:20; Developmental Studies Hybrydoma Bank, Dr. T. Jessel) that recognizes a 25-kDa truncated rat Islet-1 protein (c-terminal Islet-1) on Western blot corresponding to the amino acids residues 178–349 [23]. **Neurofilaments (NF)** with their three subunits: light (68–70 kDa), medium (145–160 kDa) and heavy (200–220 kDa) have been detected in RGC bodies and/or processes [25]. To detect the heavy, medium and light neurofilament subunits we used mouse monoclonal antibodies **anti-NF200** (1:1000; N0142, clone N52; Sigma-Aldrich, Inc.), **anti-NF160** (1:40; N5264, clone NN18; Sigma-Aldrich, Inc.), and **anti-NF68** (1:400; N5139, clone NR4; Sigma-Aldrich, Inc.), respectively. **Microtubular-associated protein-2 (MAP2)** is present in RGCs and a subset of amacrine cells [26]. For the detection of MAP2, we used a mouse monoclonal anti-MAP2 (2a + 2b) antibody (1:1000; BYA6588-1, clone AP-20; Accurate). **NeuN** expression in the retina is restricted to RGCs and amacrine cells [27]. In the current study, we used the monoclonal mouse anti-NeuN antibody (1:500; MAB377, clone A60; Millipore). **γ-Synuclein** (**γ-Syncl**) is expressed in most retinal ganglion cells, but not in other cells of the adult mouse retina [28]. We used a polyclonal goat anti γ-Synuclein antibody (1:100; sc-10698, clone E-20; Santa Cruz Biotechnology). A polyclonal goat anti-**ChAT** antibody (anti-choline **acetyl transferase**, 1:100; AB144P, Millipore) was used to visualize the two plexi of cholinergic amacrine cells. The appropriate secondary antibodies were conjugated either to Alexa Fluor 488 (Invitrogen, La Jolla, CA), Cy3 (Dianova, Hamburg, Germany), Cy5 (Dianova, Hamburg, Germany) or DyLight 649 (Jackson Immunoresearch, West Grove, PA).

### Microscopy

Retinas were examined using a Zeiss Axio Imager Z1 microscope (Zeiss, Jena, Germany) equipped with epifluorescence and a Zeiss ApoTome oscillating grating in the epifluorescence beam, which resulted in a significant reduction of out-of-focus stray light. Black and white images were taken by using a cooled CCD-camera (AxioCam MRm; Zeiss); pictures were pseudo-colored for the different fluorescent filters and superimposed using the Zeiss Axio Vision 4.2 software. Images were taken with Plan-Apochromat 20x/0.8 M27 and EC, Plan-Neofluar 40x/0.75 M27 objectives as stacks of 10 to 25 optical sections (Z-axis step size between 0.8 and 1.1 µm). To ensure a complete coverage of the retina, samples were taken at different eccentricities in each quarter of the retina wholemount.

### Analysis of the photomicrographs

Photomicrographs were analyzed through the whole z-stack, which spanned the nerve fiber layer and the inner nuclear layer (INL). For cell density measurements, all melanopsin-positive cells were counted without allocation to a specific cell type because the discrimination of cell types was not feasible in retinas of newborn animals. The density was separately calculated for each photomicrograph and then averaged for the whole age group. Cell densities are given as the mean number of cells per mm^2^ ± SEM. The measurement of soma size was performed in adult retinas on melanopsin-positive cells of clearly defined types. The soma size was measured using the Zeiss Axio Vision 4.2 software. For each cell the mean value between long and short axis were computed and taken for further calculations. The cell diameter was given as median value. For colocalization analysis, melanopsin-positive cells and cells double-labeled with a given marker were counted in different retinal locations. Cell counts from different retinae were pooled together and the percentage of marker-positive cells was calculated. For image analysis, brightness and contrast were adjusted by using the Zeiss Axio Vision 4.2 or Adobe Photoshop CS (Adobe Systems, San Jose, CA). All statistic measurements and calculations were performed with SigmaStat 3.1 (Systat Software, Inc., San Jose, CA). Statistical comparisons were done using Student’s t-test and Mann-Whitney Rank Sum test.

### Multielectrode recordings

The eyes of previously dark adapted newborn animals (P0 – P3) were enucleated under dim red light and placed in oxygenated Ames medium (Sigma-Aldrich) at room temperature. The retinas were isolated and divided into quarters, laid on a nitrocellulose membrane (Millipore) carrier and then placed ganglion cell layer (GCL) down onto nitrocellulose-coated multielectrode arrays (MEA, Multi Channel Systems, Reutlingen, Germany). MEAs consisted of sixty planar electrodes of 30 µm diameter arranged in an 8 x 8 grid with an inter-electrode distance of 200 µm forming a recording field of about 1.4 x 1.4 mm. Retinas were continuously superfused with oxygenated Ames medium at a flow rate of 1 ml/min at 35 °C. Extracellular voltage signals were processed with a MEA-1060 recording system (Multi Channel Systems, Reutlingen, Germany) as previously described [29]. Spike events were amplified x 1200, digitally filtered with a 300 Hz high-pass filter (Butterworth 2^nd^ order filter) and sampled at 32 kHz on all 60 channels simultaneously. Extracellular potentials were detected by using a threshold-based algorithm of the MC_Rack software (Multi Channel Systems, Reutlingen). Action potentials exceeding a defined voltage threshold 4.5 times the standard deviation of the baseline were digitized and stored for each negative-slope event as time-stamped spike cut-outs on the hard disk [30].

### Light stimulation

Retinas were stimulated with a Luxeon K2 high-power LED (Conrad Electronics, Hirschau, Germany). LEDs possess a relatively narrow bandwidth of 20–30 nm and have very fast onset and offset times. Since there is no LED emitting at the peak wavelength of the melanopsin photopigment (480 nm), we used a Luxeon LXHL-NE98 Star Cyan LED with a maximum emission at 505 nm. Photon flux density was measured with a calibrated UDT UV100 photodiode (United Detector Technology, San Diego, CA, USA) or by using an ILT1400A radiometer/photometer and a calibrated, broad band silicon detector (model No. SEL033; both from International Light Technology, Peabody, MA, USA). The spectral sensitivity at the peak of the melanopsin photopigment (480 nm) is only 8% higher than at the emission peak of the 505 nm LED. These relative differences amount to just 0.096 log photons * cm^-2^ * s^-1^ showing that the melanopsin photopigment is almost optimally stimulated by the LED despite the difference in the peak wavelength. Full-field light stimulation of retinal wholemounts was provided by a LED with Batwing optics positioned directly below the MEA. The LED was connected to a current source which was controlled by a STG 1004 stimulus generator (Multi Channel Systems, Reutlingen). The software MC_Stimulus (Multi Channel Systems, Reutlingen) was used to program the intensity and duration of the light stimulation. Rectangular pulses of varying voltages were used to adjust light intensity. Most stimuli were repeated threefold interrupted by a 5 min dark pause between them for recovery of the photopigment. In the flicker experiments, stimuli were repeated 10 fold interrupted by varying pauses. The time of light stimulation was monitored by MC_Rack software.

### Analysis of electrophysiological data

Spikes from individual ipRGCs were discriminated off-line. Briefly, MC_Rack data files were exported into Spike2 software (Spike2 software V.7; Cambridge Electronic Design, CED, Cambridge, UK) and spikes from individual neurons were separated by spike sorting algorithms and principal component analysis when necessary. Spike sorting was only performed when spike waveform and amplitude clearly distinguished more than one unit on an electrode; otherwise we analyzed multiunit responses without spike sorting. Electrodes that showed a clear increase (> 100%) of the spike rate during each light pulse compared to the activity recorded in darkness were taken as light responsive and used for analysis of irradiance-response curves. Four parameters were analyzed to characterize light responsive cells: 1) latency to first spike, 2) latency to peak firing rate, 3) response duration, and 4) peak firing rate. All data were averaged between the individual cells, normalized for each parameter and fitted with a sigmoidal Michaelis-Menten equation [31–33].

### Pharmacological treatment

For each preparation any potential input from rod and cone photoreceptors to ipRGCs was suppressed in initial control experiments by bath application of metabotropic and ionotropic glutamate receptor agonists and antagonists. L-AP4 (*L*-(+)-2-amino-4-phosphonobutyric *acid*; 100 µM) blocked signal transfer at group III metabotropic glutamate receptors; NBQX (2,3-dioxo-6-nitro-1,2,3,4*-tetrahydrobenzo[f]quinoxaline-7-sulfonamide*; 40 µM) blocked selectively AMPA receptors and D-AP5 (*D-*(*-*)*-2-amino-5-phosphonopentanoic acid*; 30 µM) NMDA receptors. The concentrations used in the present study were sufficient to block any potential signal transfer from outer photoreceptors onto ipRGCs in retinas of adult *Arvicanthis* . In young postnatal animals (P0 – P8), where functional input from rods and cones onto RGCs is lacking, there was no obvious difference in the response pattern with or without blocking cocktail. In most recordings, no attempt was made to block the waves of correlated activity occurring in *Arvicanthis* in the developing inner retina, thus proving the intrinsic nature of the light responses observed in ipRGCs. In MEA recordings, spontaneous retinal waves sweep across neighbouring electrodes and are easily distinguishable from light-evoked responses. All drugs were purchased from Biotrend (Köln, Germany).

## Results

### Different morphological types of ipRGCs in the *Arvicanthis* retina

**Figure 1:**
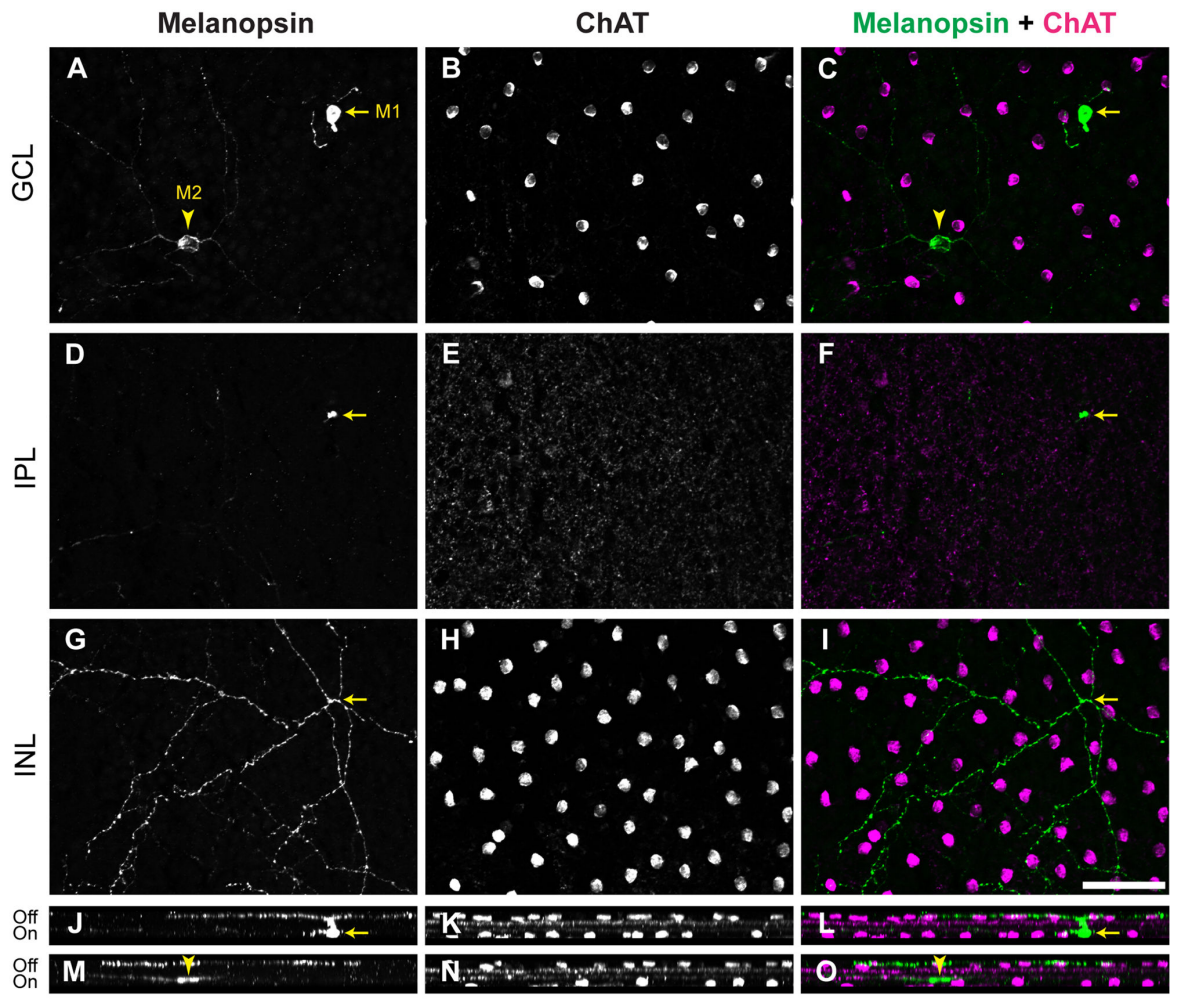
Morphology of M1 and M2 ipRGCs in the retina of *Arvicanthis.* **A**–**I**) Wholemounts of the retina from adult (9 months old) *Arvicanthis* immunostained for melanopsin (A, D, G; green in C, F, I) and ChAT (B, E, H; magenta in C, F, I), a cholinergic amacrine cell marker. Pictures were taken from focal planes on the GCL (A–C), IPL (D–F) and IPL/INL border (G–I). M1 cells show an intense melanopsin staining (arrow in A and C). Strongly melanopsin-positive dendrites of M1 cells pass the IPL (arrow in D and F) and stratify in the outer portion of the IPL (OFF sublamina) in proximity to the INL (in G and I the arrow shows the branching point of the main dendrite); M2 cells show a less intense melanopsin staining (arrowhead in A and C). Weakly melanopsin-positive M2 dendrites stratify in the inner portion of IPL (ON sublamina) near to the GCL (A–C). **J**–**L**) Side view and stratification level of M1 cell (soma indicated by an arrow). **M**–**O**) Side view and stratification level of the M2 cell (soma indicated by an arrowhead); note that in (M) and (O) also the intense OFF plexus of processes from melanopsin cells is visible. ChAT, choline acetyl transferase; GCL, ganglion cell layer; IPL, inner plexiform layer; INL, inner nuclear layer; Off, OFF sublamina of IPL; On, ON sublamina of IPL. Scale bar: 50 µm.

**Figure 2:**
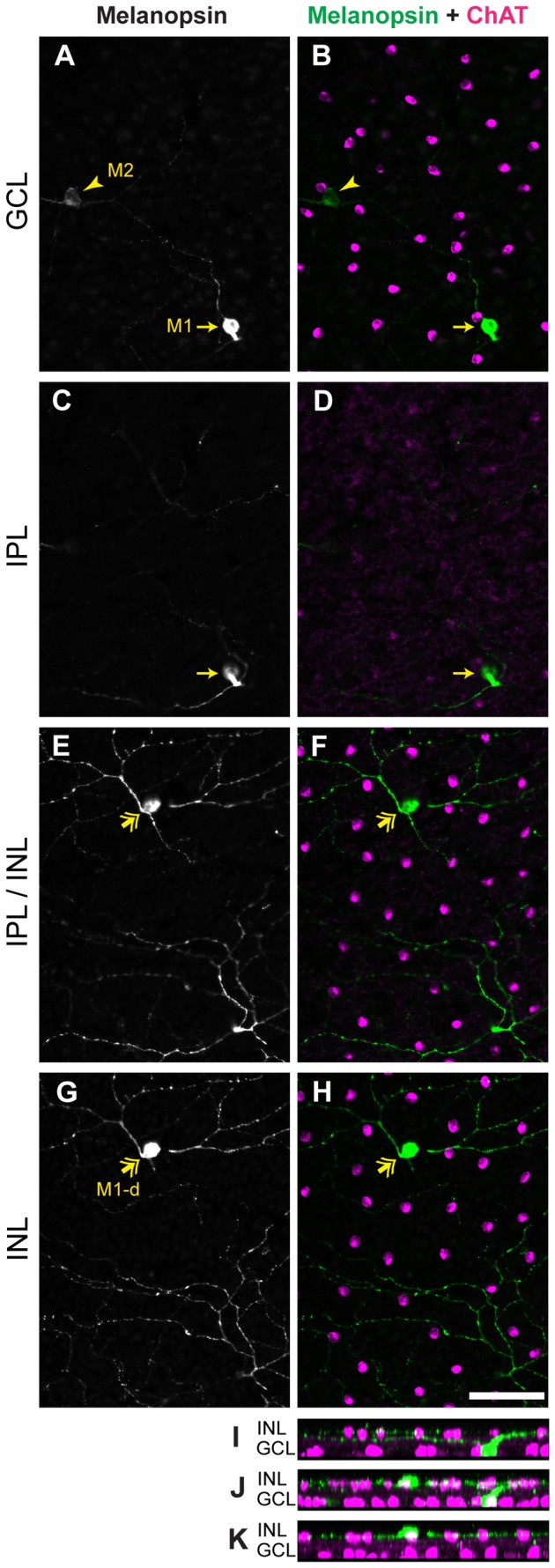
Morphology of orthopic and displaced ipRGCs in the retina of *Arvicanthis.* **A**–**H**) Retinal wholemounts of adult (9 months old) *Arvicanthis* immunostained for melanopsin (A, C, E, G; green in B, D, F, H) and ChAT (magenta in B, D, F, H), a cholinergic amacrine cell marker. Pictures were taken from focal planes on the GCL (A–B), IPL (C–D), IPL/INL border (E–F) and the INL (G–H). **I**–**K**) Side view of the cells. Orthotopic M1 cell (arrow in A and C) have cell bodies located in the GCL and stratify in the outer portion of the IPL (OFF sublamina), close to the INL (E–H and I). These cells show stronger melanopsin-immunoreaction than M2 cells (arrowhead in A), including cell body and dendrites. Displaced M1 (M1-d) cells show a comparable intensity of melanopsin staining to the orthotopic M1 cells, nevertheless their cell bodies are located in the INL (two headed arrow in E and G). M1-d cells stratify in the outer portion of the IPL, similar to their orthotopic counterparts (E–H and J–K). Figure 2 I shows only one M1 cell, Figure 2 J a M1 and a M1-d cell, and Figure 2 K only a single M1-d cell. ChAT, choline acetyl transferase; GCL, ganglion cell layer; IPL, inner plexiform layer; INL, inner nuclear layer. Scale bar: 50 µm.

Melanopsin-expressing ipRGCs can be classified in several subtypes, based on the location of their somata, the soma size, the intensity of melanopsin staining and their stratification patterns of dendritic arborizations. Co-staining with an antibody to choline acetyltransferase (ChAT), which reliably stains somata and dendrites of cholinergic amacrine cells, served as a marker to distinguish ON and OFF sublaminae of the inner plexiform layer (IPL) and ensured the exact localization of the ipRGCs in the retinal layers. The majority of ipRGCs (2063 of 2787 ipRGCs; 74%) were strongly melanopsin-immunopositive with somata located in the GCL and arborizations in the outermost part of the IPL (OFF sublamina) (Figures 1 and 2). These cells, classified as M1 type, had small to medium sized somata: 8.5–19.5 µm in diameter (median size 13 µm, n = 541) (Table 1).

**Table 1:** Somatic diameter for different ipRGC types in the retina of *Arvicanthis*.

**Cell Type**	**n**	**Min**	**Max**	**Median**	**Q1 (25%)**	**Q3 (75%)**	**Mean**	**SD**
M1	541	8.5	19.5	13	12	14.3	13.2	1.7
M2	201	10.3	21.2	15.2	14.1	16.3	15.2	1.9
M-d	20	9.6	14	11.9	11.2	12.9	11.9	1.2

Descriptive statistics of the soma diameter [in µm] for the three ipRGCs types in the retina of *Arvicanthis* . There is a statistically significant difference in soma diameter between the different groups (one way ANOVA; p<0.001).

The second class, summarized in the mouse retina as non-M1 ipRGCs, consists of several subtypes (M2 – M5) [34]. In *Arvicanthis* , this population mainly expressed features of the M2 type, characterized by somata located in the GCL and dendrites branching in the innermost layer (ON sublamina) of the IPL (Figures 1 and 2). The somata ranged from 10.3 to 21.2 µm (median size: 15.2 µm, n = 201), and the melanopsin immunolabel was weaker than in M1 cells. The M2 type constituted ~25% (700 of 2787) of all melanopsin-positive ipRGCs in *Arvicanthis* . Due to their faint staining with the melanopsin antibody, the other types (M3 – M5), previously described in the mouse retina, could not be reliably identified in *Arvicanthis* *.*

A few ipRGCs possess somata displaced to the inner nuclear layer. In the mouse retina displaced cells were shown to comprise M1-like displaced and M2-like displaced ipRGCs [20,35]. In *Arvicanthis* , all displaced cells (termed M-d cells) were strongly melanopsin-immunoreactive and stratified in the outer IPL, similar to M1 ipRGCs (Figure 2). The somata of M-d cells were 9.6–14 µm in diameter (median value 11.9 µm, n = 20). M-d cells were very rare and constituted only ~1% ipRGCs compared to ~6% in the mouse retina [20]. Because of the clear stratification in the OFF-sublamina of the IPL and the strong melanopsin immunoreactivity, all displaced cells in *Arvicanthis* seem to be of the M1-like type (M1-d). They could be easily distinguished from orthotopic M1 cells and were considered separately.

In general, the morphology of the different types of melanopsin-immunopositive cells in the retina of diurnal *Arvicanthis* closely resemble those described previously in nocturnal rodents [20,36–38]. However, the respective proportions of each subtype were significantly different, with a high percentage of M1 cells (74%) in *Arvicanthis* compared to only 44% in the mouse retina [20]. It is possible that these differences may be partly caused by antibody specificity. As the antibody against the N-terminus of mouse melanopsin (UF006) does not cross-react with species outside mouse (I. Provencio, pers. comm.), we used only the antibody recognizing the melanopsin C-terminus (Pa1-781), which gave robust staining in all preparations. Since melanopsin exists in two isoforms, Opn4L and Opn4S, with divergent C-terminal sequences and which show differential expression in ipRGCs [39], antibody specificity may influence the observed patterns.

### Comparison of ipRGC density in the retina of *Arvicanthis* during postnatal development

**Figure 3:**
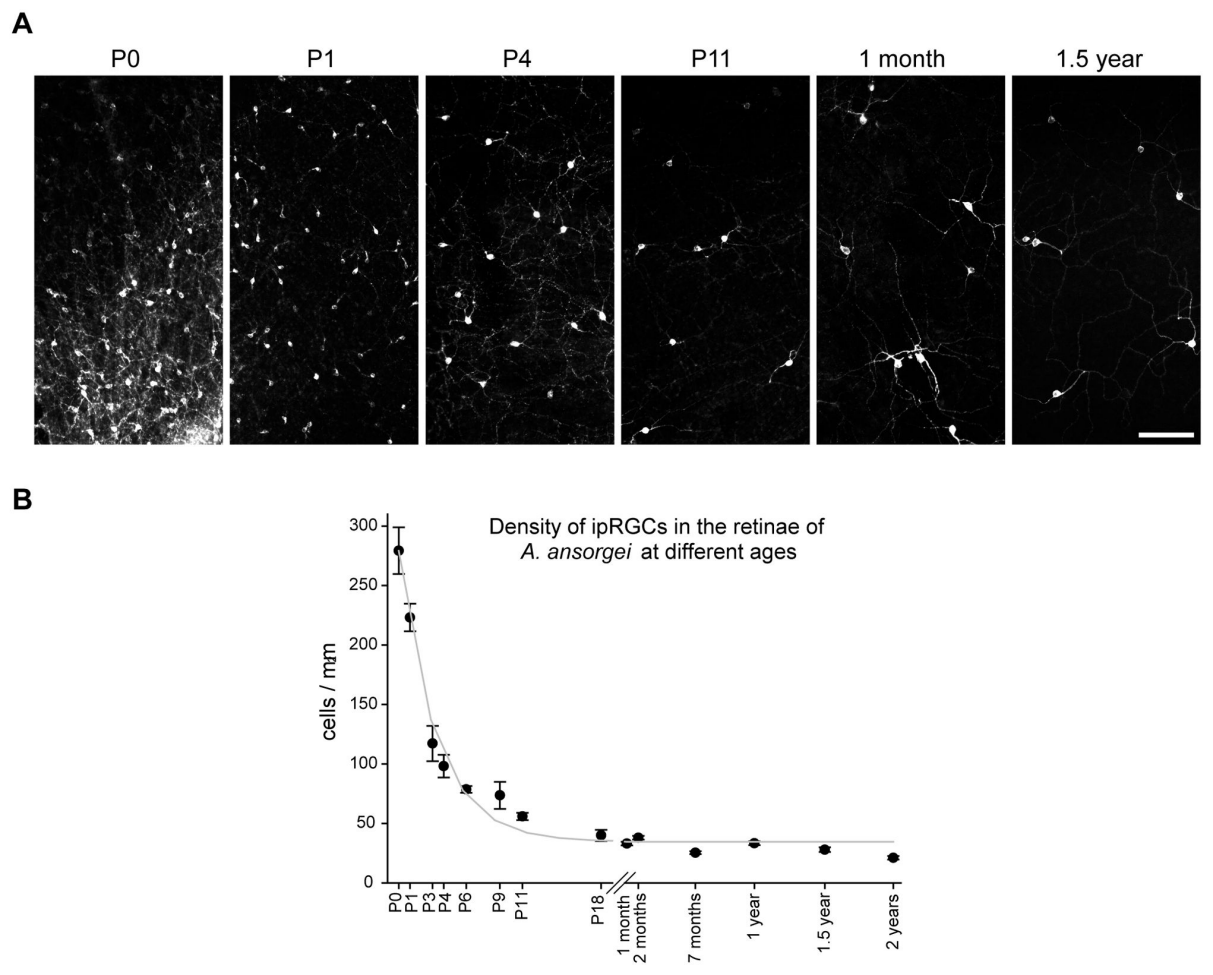
Density of melanopsin-positive ipRGCs in the retina of *Arvicanthis* at different ages. **A**) Representative photomicrographs showing the change of the density of melanopsin-positive ipRGCs at different stages of the postnatal development. Scale bar: 100 µm. **B**) Plot showing a change of mean density of ipRGCs in the course of postnatal development. In the new-born retina (P0, postnatal day 0) almost 300 cells/mm^2^ were observed. The values considerably declined during the first 2 postnatal weeks reaching a density of 20–40 cells/mm^2^ in adult animals and stayed at nearly constant levels until the late adulthood. Values are presented as mean density (cells/mm^2^) ± SEM (1–6 retinae per age group).

During postnatal development, ipRGCs were observed throughout the retina of P0 Arvicanthis, the earliest period investigated in the present study. Melanopsin was expressed in cell somata and processes, but in newborn pups the dendrites did not yet stratify into the two characteristic plexi seen in adult retina. In the first postnatal week the plexi gradually grew into the two strata, and could be distinguished clearly in wholemounts by the second week (~P9). The lack of clear stratification in the early postnatal stages made it difficult to distinguish the different ipRGC subtypes. However, using Brn3 expression as a guide (see below), the ratio of M2 to M1 type appeared to be different than in adult, with lower percentage of M2 cells in newborn retinas, since Brn3 expression was seen in ~5% ipRGC at P0 (compared to ~19% in adult retina). For density measurements we scored total numbers of all ipRGCs, irrespective of their type. We analysed 34 retinas from different animals aged between P0 and 26 months. At P0 the density was 280 ± 19.6 cells/mm^2^ across the retina. This number dropped significantly to 74 ± 11.3 cells/mm^2^ at age P9, and declined further to 33 ± 1.6 cells/mm^2^ by 1 month, after which it remained relatively constant (2 year old: 21 ± 1.5 cells/mm^2^) (Figure 3A, B). In general, there is a 10-fold drop in the ipRGCs density during postnatal development of the *Arvicanthis* retina. Considering the growth of the eye during postnatal development (total retinal area at P0: 13.9 ± 0.6 mm^2^; at P9: 33.4 ± 5.5 mm^2^ and in 8 month to 1 year old animals: 60.9 ± 2.4 mm^2^), the estimated number of ipRGCs reached almost 4000 cells in newborn animals, and declined then to about 2500 cells at P9 and finally to 1800 cells in adult *Arvicanthis* .

### Molecular analysis of ipRGCs in the retina of *Arvicanthis*

**Figure 4:**
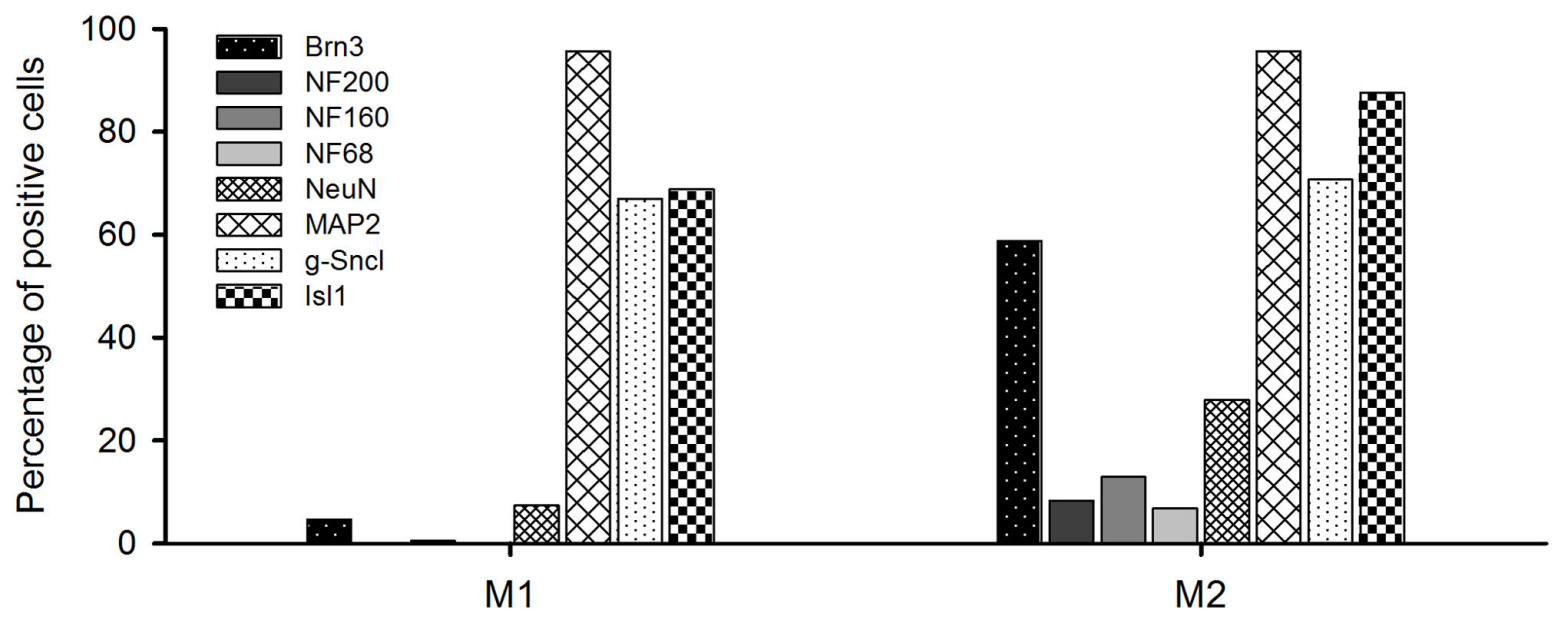
Distribution of ipRGCs in the retina of *Arvicanthis* immunopositive to specific RGC markers. Melanopsin-positive ipRGCs of both types (M1 and M2) show high expression of MAP2, γ-Syncl and Isl1. Brn3 distinguishes between M2 and M1 cells. Almost 60% of the M2 type expresses Brn3, but only 5% of the M1 type. Another clear difference between M1 and M2 cells is observed in the expression of different neurofilaments (NF200, NF160 and NF68) that label a small population of the M2 cells, but virtually none of the M1 ipRGCs. Expression of NeuN is higher in the M2 type than in the M1 cells.

**Figure 5:**
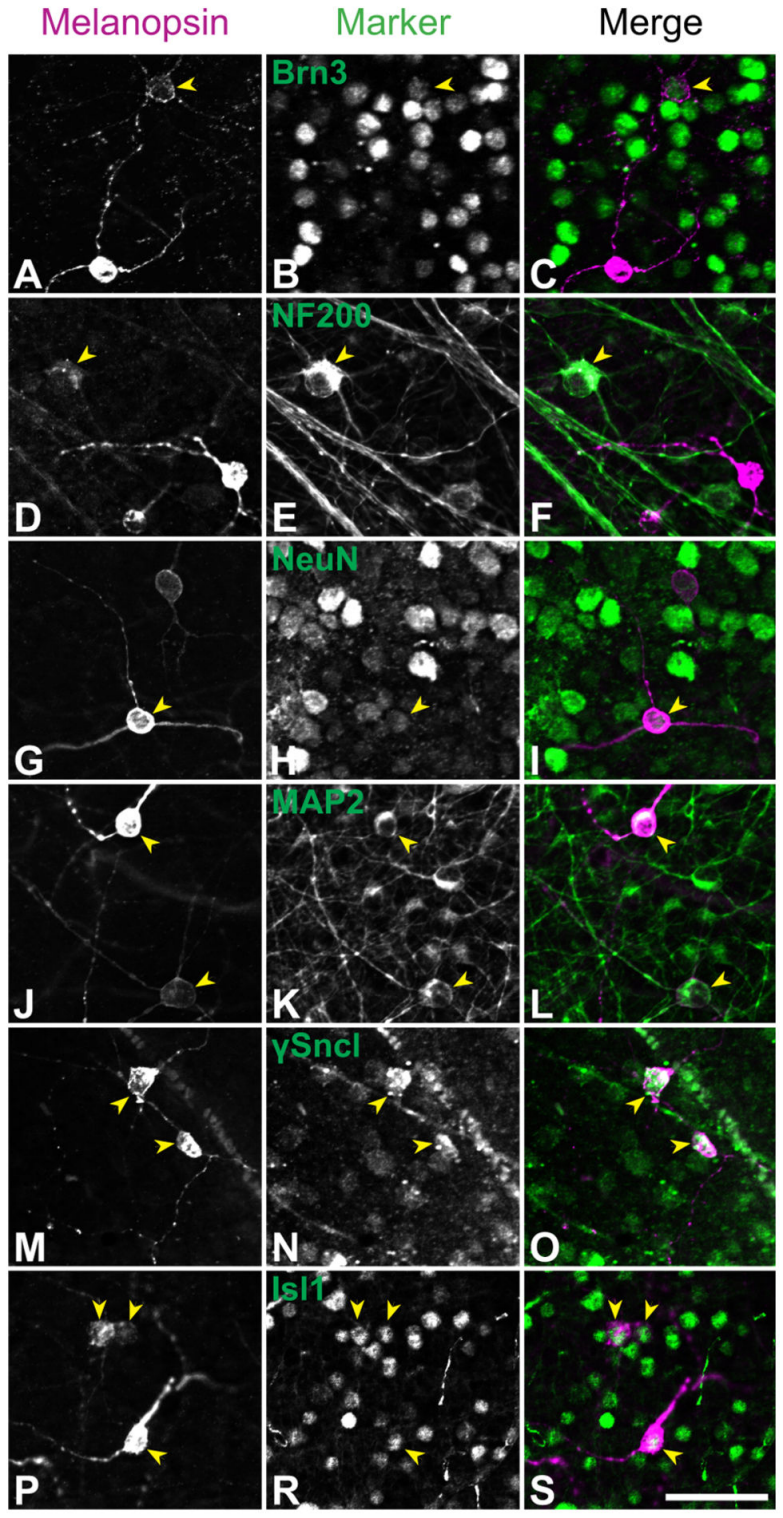
Expression of different RGC markers in retinal ipRGCs of *Arvicanthis*. Double immunolabeling of retina wholemounts with an anti-melanopsin antibody (left column) and a specific RGC marker (middle column). The right column depicts the superposition of the two previous pictures (melanopsin-positive ipRGCs in magenta, specific markers in green). Cells showing a colocalization of melanopsin and the given marker are indicated by yellow arrowheads. Scale bar: 50 µm. **A**–**C**) Colocalization of Brn3 and melanopsin in ipRGCs. Brn3 is expressed only in some ipRGCs, mostly of the M2 group. The picture shows one M2 cell expressing Brn3, the other ipRGC (M1) is negative to Brn3. **D**–**F**) Colocalization of NF200 and melanopsin in ipRGCs. Only very few ipRGCs express NF200 and all of them belong to the M2 cells. The NF200+-ipRGCs have large somata and show very faint melanopsin-immunoreactivity. **G**–**I**) Colocalization of NeuN and melanopsin in ipRGCs. NeuN is expressed only in some M1 and M2 ipRGCs. One NeuN-positive M1 ipRGC is shown in the picture, the other melanopsin+-RGC (M2) is negative to NeuN. **J**–**L**) Colocalization of MAP2 and melanopsin in ipRGCs. Nearly all melanopsin+-RGCs coexpress MAP2. The picture shows one M1 and one M2 ipRGCs coexpressing MAP2. **M**–**O**) Colocalization of γ-Synuclein (γ-Sncl) and melanopsin in ipRGCs. γ-Synuclein is expressed in the majority of M1 and M2 ipRGCs. Two γ-Synuclein+ M1 cells are shown in the picture. **P**–**S**) Colocalization of Isl1 and melanopsin in ipRGCs. The great majority of M1 and M2 ipRGCs express Isl1. In the picture two M2 and one M1 cells are visible, all show Isl1-immunoreactivity.

For a more detailed characterization of the different ipRGC types, we performed double immunostaining using an anti-melanopsin antibody and a number of antibodies directed against proteins typically expressed in RGCs. The results summarizing the colocalization of the different RGC markers with melanopsin are presented in Table 2 and Figure 4. All ipRGC types in *Arvicanthis* retina showed broad expression of MAP2 (95% of 231 ipRGCs), γ-Synuclein (68% of 145 cells) and Isl1 (75% of 272 cells), whereas Brn3, the different neurofilament subunits (NF200, NF160 and NF68) and NeuN were only found in a small subset of cells (Brn3: 19%, NF200: 2%, NF160: 3%, NF68: 2%, NeuN: 12%). Double immunofluorescence labeling for melanopsin and the different markers are shown in the photomicrographs in Figure 5.

**Table 2:** Expression of RGC markers in ipRGCs of *Arvicanthis.*

	Percentage of ipRGCs positive to the marker and total number of marker-positive cells		
**Marker**	**All ipRGCs**	**M1**	**M2**
**Brn3**	19.1 % [403]	4.6 % [70]	58.8 % [332]
**NF200**	2.2 % [14]	0	8.3 % [14]
**NF160**	2.9 % [15]	0.5 % [2]	12.9 % [13]
**NF68**	2.0 % [10]	0	6.8 % [10]
**NeuN**	11.8 % [23]	7.4 % [11]	27.9 % [12]
**MAP2**	94.8 % [219]	95.6 % [175]	95.7 % [44]
**γ-Synuclein**	68.3 % [99]	67.0 % [69]	70.7 % [29]
**Isl1**	75.4 % [205]	68.6 % [120]	87.6 % [85]

Summary of the expression of eight different retinal ganglion cell (RGC) markers. The cells from different retinas were analysed, the data pooled and the percentage of positive cells was calculated for the different ipRGC types. Additionally, the total number of marker-positive cells in each group is indicated in square brackets. 

#### M1 cells

Virtually all ipRGCs of the M1 type expressed MAP2 (96%), and many expressed Isl1 (69%) and γ-Synuclein (67%). However, NeuN and Brn3 stained only small numbers of M1 ipRGCs (7% and 5%, respectively), and labeling with the different neurofilaments was almost completely absent (only 2 of 414 cells for NF160, no NF200- or NF68-positive cells).

#### M2 cells

The M2 cell type showed a similar broad expression of MAP2 (96%), Isl1 (88%) and γ-Synuclein (71%), whereas NeuN, Brn3 and the three different neurofilaments were present in higher numbers in M2 compared to M1 types (Table 2). The most prominent difference was observed in Brn3 expression: ~60% of M2 cells were Brn3-positive. Neurofilament expression was still low in M2 type ipRGCs, but significantly higher than in M1 cells (8.3% NF200^+^, 12.9% NF160^+^ and 6.8% NF68^+^).

#### Displaced cells

The number of displaced ipRGCs was extremely low in *Arvicanthis* retinas (only 24 of 2787 ipRGCs, ~1%) and we could not examine expression of the different markers.

The Brn3 marker exhibits a high affinity to M2 ipRGCs and differentiates between the M1 and M2 subtypes; more than 80% of Brn3-positive ipRGCs in adult retinas are M2 cells. Thus, we estimated the distribution of ipRGCs subtypes in newborn *Arvicanthis* through testing the Brn3 expression in P0 animals. We found that only 5.3% of all ipRGCs expressed Brn3 (data not shown). In comparison with adult percentage (19.1%), these results point to a lower M2/M1 ratio in newborn vs. adult retina.

### Physiology

**Figure 6:**
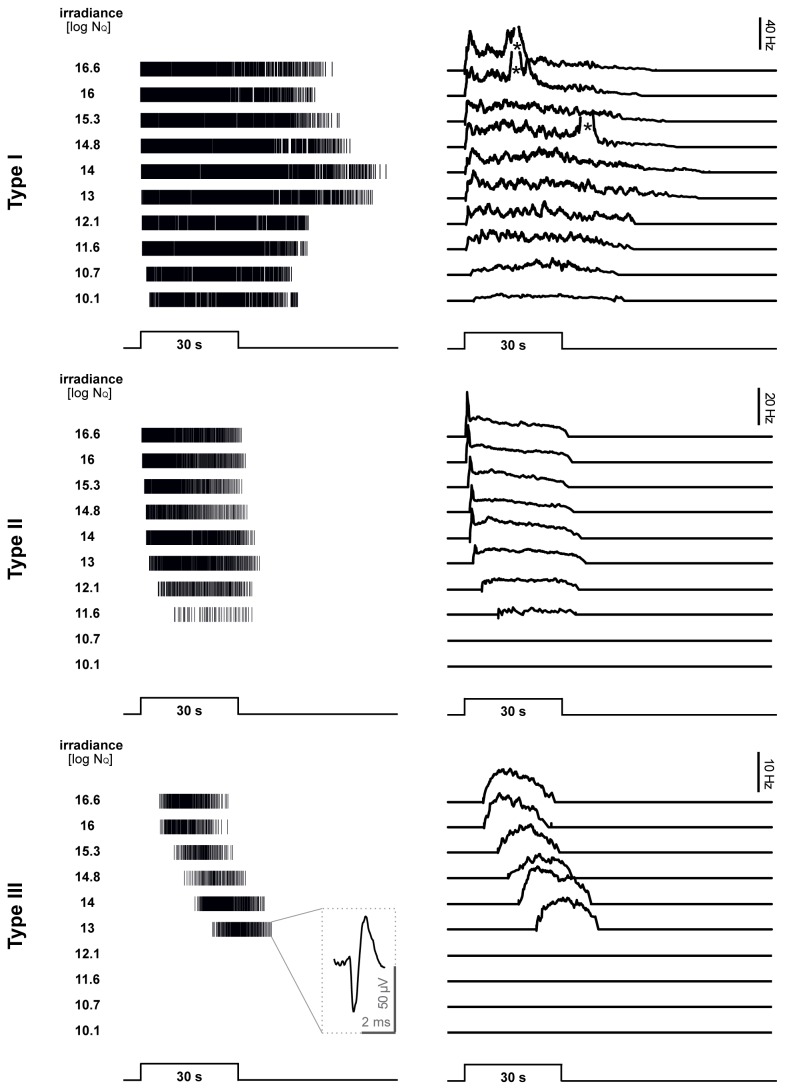
Physiological heterogeneity of ipRGCs in newborn *Arvicanthis* retinas. Light evoked responses of the three ipRGC types to 30 s stimuli (505 nm, full-field illumination) of increasing intensity recorded from a P0 Arvicanthis retina. Left column shows representative examples of spike trains recorded with multielectrode arrays. Each vertical line represents a single action potential. The inset in the left column in the lower graph shows a magnification of such an action potential. Right column depicts the corresponding histograms of the firing rate. The asterisks in the right column point to retinal waves that interfere with the spike responses of ipRGCs. Upper graphs ipRGCs type I, middle graphs type II, and lower graphs type III.

**Figure 7:**
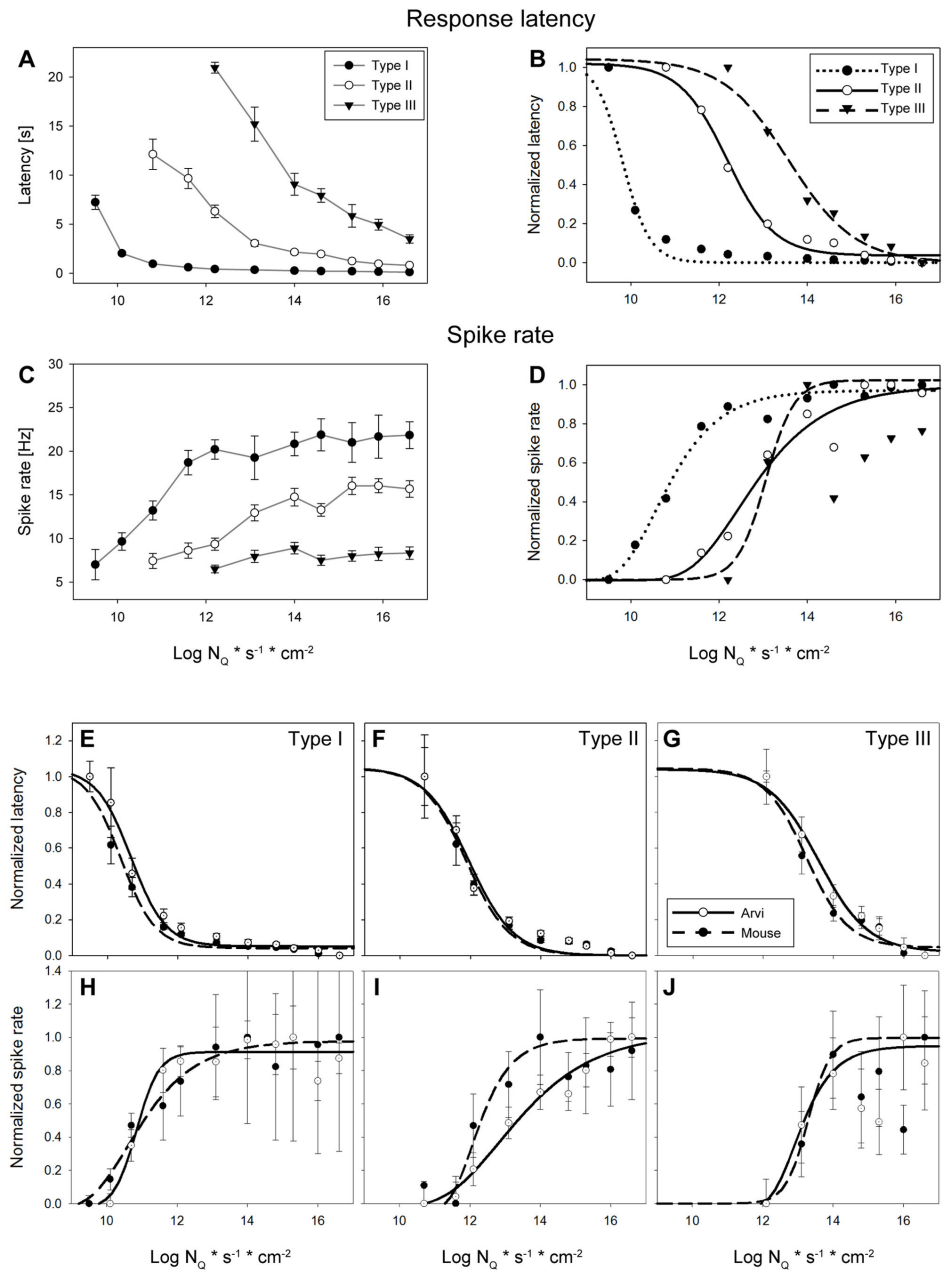
Response characteristics of the three ipRGC types recorded from newborn *Arvicanthis* and mouse retinas. **A**–**B**) response latency and C–D**)** light-evoked spike rate. (A) and (C) depicts the mean values (± SEM) and (C) and (D) the normalized response fitted with a sigmoidal Michaelis-Menten function. Light responses of single units measured from P0 – P3 retinas (4 retinas, n = 21, 51 and 21 for types I, II and III, respectively). Stimulus: 505 nm, 30 s. **E**–**J**) Response characteristics of the different ipRGCs types from newborn *Arvicanthis* (open circles, black curves) in comparison to ipRGCs from retinas of newborn C57BL/6 mice (filled circles, dashed curves). *Arvicanthis*: n = 93 cells (4 retinas; P0 – P3); mice: n = 39 cells (3 retinas; P0 – P2). **E**–**G**) Normalized response latency for cells of type I (E), II (F) and III (G). **H**–**J**) Normalized spike rate of type I (H), II (I) and III (J). All values are mean ± SEM. Stimulus: 505 nm, 30 s.

MEA recordings of ipRGCs from retinas of newborn (P0 – P3) *Arvicanthis* showed three different response types distinguished by their sensitivity to light, response strength, latency, and duration. Examples of light responses with their typical patterns are shown in Figure 6 as spike trains (left side) and the corresponding rate histograms (right). Figure 7A–D depicts the intensity-response curves for the response latency and spike rate of each ipRGC type in *Arvicanthis* . As we could not provide a direct link between physiological and morphological types in *Arvicanthis* , we based the nomenclature of the different ipRGC types on previous studies showing the highest light sensitivity in M1 ipRGCs [37] (our physiological type I) and classified the other types according to their sensitivity. Type I cells were the most sensitive, with very short latencies to activity onset, high spike rates and prolonged response durations. Type II ipRGCs were easily recognized because of their large action potential amplitude. This type was the most frequent, and was characterized by intermediate latencies and durations, with a spike rate and response duration being lower than in type I cells. The third response type (type III) showed low photosensitivity, very long latencies and short response duration. Among a sample of 93 completely analyzed ipRGCs we observed 20% type I, 59% type II and 21% type III cells.

Type I ipRGCs responded rapidly to light stimulation. Latencies to activity onset were between 7.2 ± 0.7 s for light stimuli just above threshold (9.5 log N_Q_ * s^-1^ * cm^-2^), up to 0.10 ± 0.01 s for saturating flashes (16.6 log N_Q_ * s^-1^ * cm^-2^) (Figure 7A), and the corresponding values for the peak latencies were 20.4 ± 2.2 s (9.5 log) and 0.68 ± 0.05 s (16.6 log), respectively. After reaching peak activity, the spike rate of type I ipRGCs remained fairly high during light stimulation (Figures 6 and 7C). With very long light stimulation, light-evoked activity persisted at high rates, but the firing pattern became irregular showing many short bursts (data not shown). At light offset, type I ipRGCs showed a very slow decay of activity. The firing rate gradually returned to baseline levels depending on the stimulus intensity. Interestingly, the response duration was longest at lower photopic levels. At high photopic levels (> 14 log N_Q_ * s^-1^ * cm^-2^), response duration (Figure 6), but not the spike activity, declined (Figure 7 C-D). Irradiance-response curves revealed that type I ipRGCs had the highest photosensitivity of all three types examined with an irradiance yielding half-maximal responses (IR 50) of 10.7 log N_Q_ * s^-1^ * cm^-2^ measured for the response latency and 10.9 log N_Q_ * s^-1^ * cm^-2^ for the spike rate, respectively (Figure 7B, D).

Under saturating light stimulation, type II cells were characterized by a sharp increase in activity ~1 s after light onset, followed by relaxation to a steady state firing rate which was maintained throughout light presentation (Figure 6). After cessation of light stimulation the firing rate rapidly returned to baseline values. At threshold intensities, type II showed a long latency to activity onset (12.1 ± 1.5 s at 10.7 log N_Q_ * s^-1^ * cm^-2^; Figure 7A), more than 10-fold longer as type I for the same intensity, and also for the strongest stimuli the response latency of type II was longer than type I (0.81 ± 0.1 s at 16.6 log N_Q_ * s^-1^ * cm^-2^). Photosensitivity (IR 50) of type II ipRGCs was in the range of 12 log N_Q_ * s^-1^ * cm^-2^ for response latency and 13.3 log N_Q_ * s^-1^ * cm^-2^ for spike rate, about 1–2 log units lower than of type I ipRGCs (Figure 7B, D).

The third response type (type III) was characterized by a long latency to activity onset and to peak activity, low firing rate and low photosensitivity (Figures 6 and 7). The threshold response latency (20.95 ± 0.56 s at 12.1 log N_Q_ * s^-1^ * cm^-2^), was ~50-fold longer than type I and 3-fold longer than type II. The photosensitivity was in the range of log IR50 = 13.4–13.6 log N_Q_ * s^-1^ * cm^-2^ (for spike rate and latency, respectively), i.e. up to 1.7 log units lower than type II and about 2.5–3 log units lower than type I (Figure 7B, D).

To have a direct comparison to a nocturnal rodent, we repeated the experiments using C57BL/6 mouse retinas under identical conditions. 39 ipRGCs were recorded from 3 newborn (P0 – P2) mouse retinas and categorized into the three physiological types. Figure 7E–J shows the comparison of response latency and firing rates for *Arvicanthis* and mouse retinas. Response latencies were almost identical between both species (Figure 7E–G), and irradiance-response curves with spike rate as response parameter also showed close resemblance. Photosensitivity of type I and type III cells exhibited comparable log IR 50 values; only type II cells showed some variations between these two species (Figure 7H–J). However, the comparison of values for the absolute spike rate reveal higher firing of all three ipRGC subtypes in *Arvicanthis* compared to the mouse (data not shown) and also a striking difference was observed in the absolute latency of type I ipRGCs. For an intensity of 10.7 log N_Q_ * s^-1^ * cm^-2^ the latency was 1.14 ± 0.2 s in *Arvicanthis* and 4.6 ± 0.6 s in the mouse. The distribution of the different physiological types in the mouse retina matched the distribution in *Arvicanthis* (type I: 23%, type II: 49% and type III: 28%).

In the mouse retina, the rod and cone based photoreceptive pathway becomes functional at about P12, when the vertical synaptic connections provided by bipolar cells are established [40]. To ensure that the photoresponse recorded from the P0 – P3 *Arvicanthis* retina had no synaptically driven component, light responses were also recorded in the presence of metabotropic and ionotropic glutamate receptor agonists and antagonists, to block any potential signal transfer from outer photoreceptors onto ipRGCs. There were no differences in the light response before and after synaptic blockade, confirming that the ipRGCs at P0 – P3 are intrinsically photosensitive without receiving input from rod or cone-driven photoresponses (data not shown).

**Figure 8:**
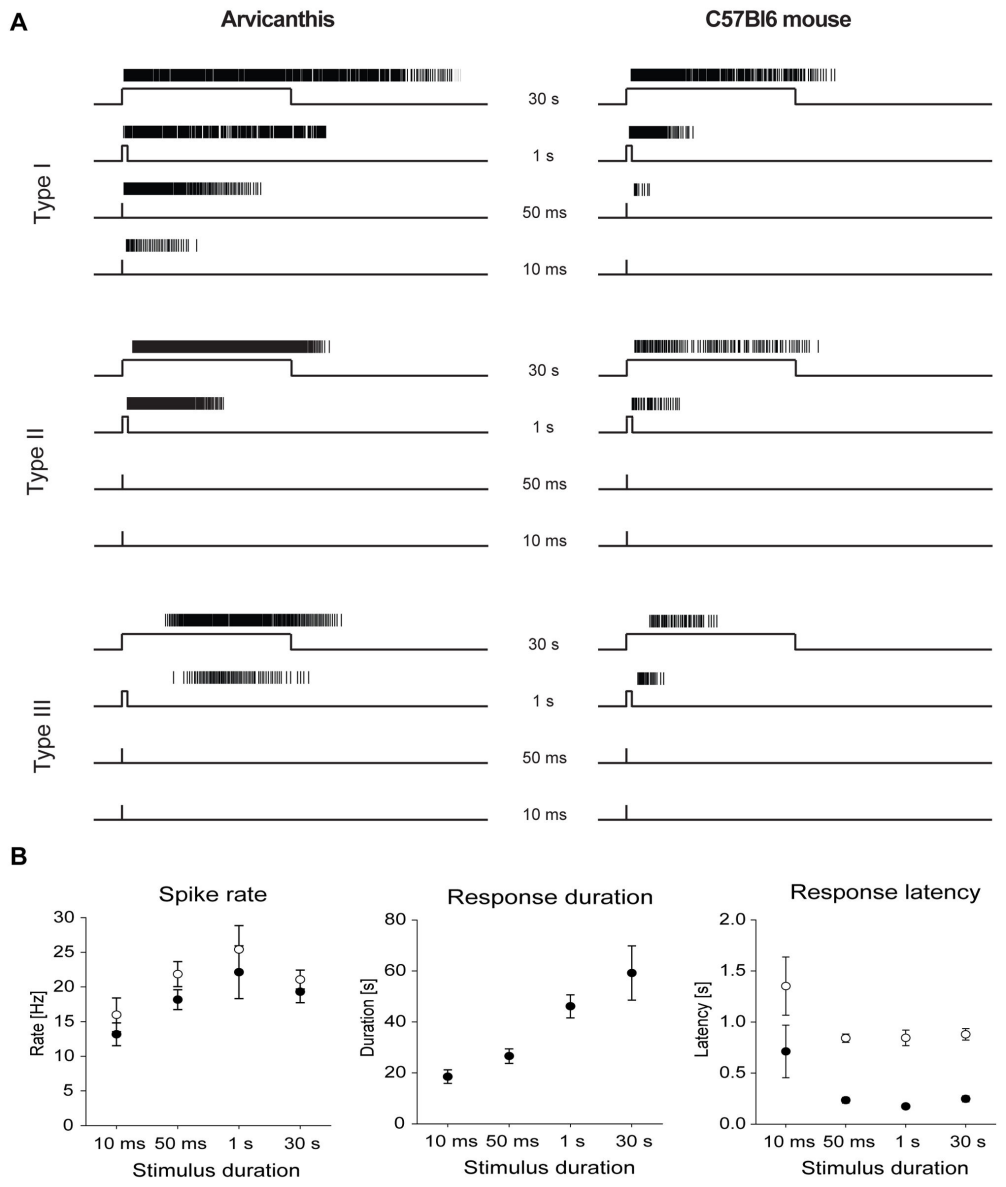
Responses of ipRGCs from *Arvicanthis* and mouse retina to stimuli of different duration. **A**) Light-induced responses of ipRGCs in *Arvicanthis* (left) and in the mouse retina (right) to short flashes of constant irradiance (14.3 log N_Q_ * cm^-2^ * s^-1^). The most sensitive ipRGC type (type I) respond to very short light flashes of up to 10 ms in *Arvicanthis* and 50 ms in the mouse, respectively, whereas type II and type III ipRGCs were insensitive to flashes shorter than 1 s. **B**) Response characteristics of ipRGCs type I of *Arvicanthis* to light flashes of different duration. The left graph shows that the mean spike rate (filled circles) and peak firing rate (open circles) reached a maximum with stimuli lengths of 1 s. The response duration (middle graph) continuously increased with the stimulus duration, whereas response latencies (right graph; filled circles: latency to the first spike, open circles: latency to peak firing rate) rapidly declined with increasing stimulus duration. Data presented as mean values ± SEM.

**Figure 9:**
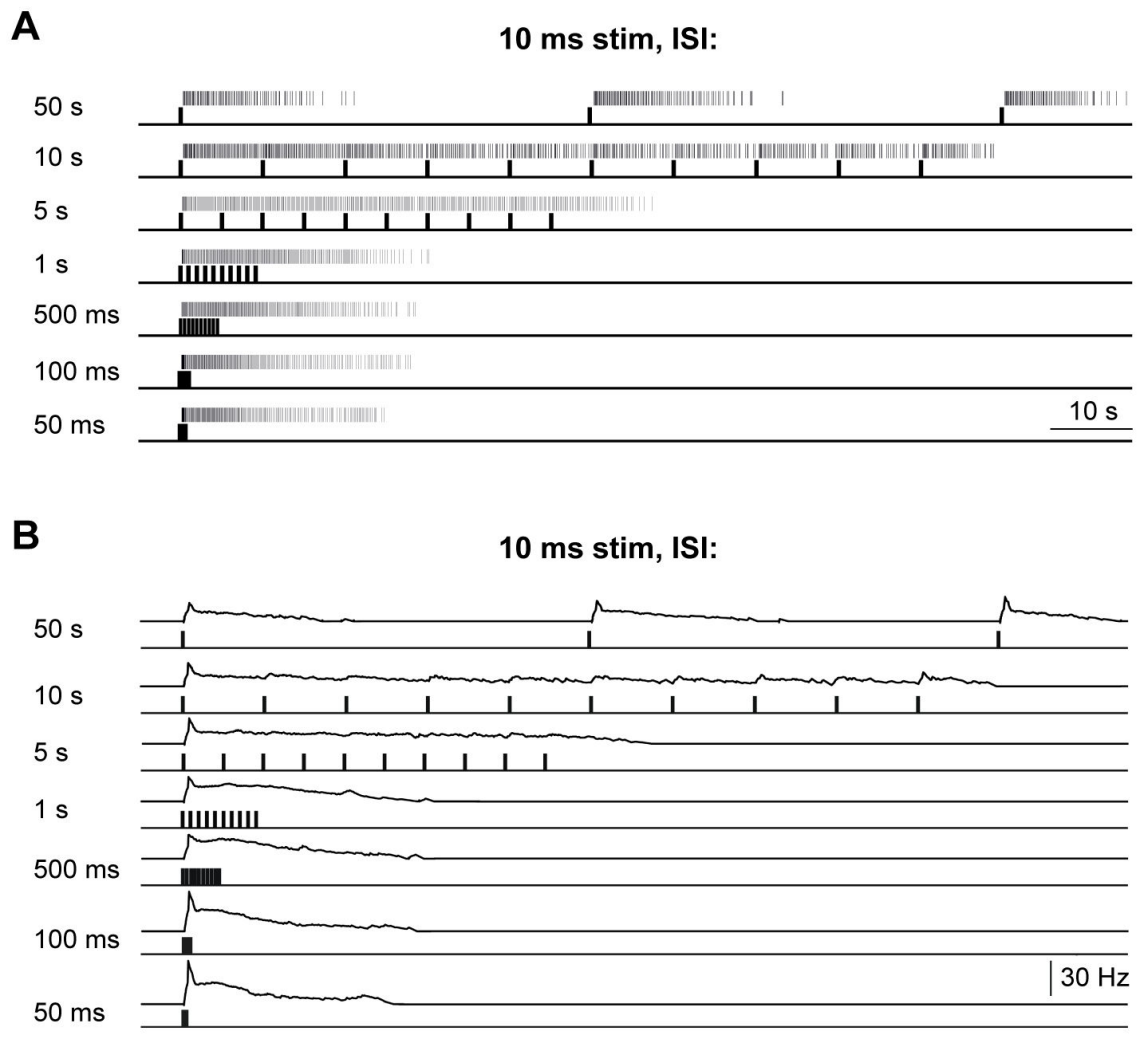
Type I ipRGCs’ response to flash stimuli (10 ms) of different frequency. Light responses of a type I ipRGCs of *Arvicanthis* to brief, repeated light stimulation of constant irradiance (505 nm; 10 ms; 14.3 log N_Q_ * cm^-2^ * s^-1^). **A**) Spike trains in response to repeated light flashes, and (**B**) corresponding spike histograms. Light stimulation is shown below the spike trains and the histograms as an upward deflection of the traces. Flashes were applied with interstimulus intervals (ISI) between 50 ms and 50 s (20–0.02 Hz) indicated on the left side. For each ISI flashes were repeated 10x. However, for the ISI of 50 s only the first three light flashes are shown. The Lomb-Scargle periodograms revealed significant periods for the 5 s, 10 s and 50 s ISI; when the time between two stimuli is below 5 s, single responses showed a fusion with the preceding response.

Intrinsic photoresponses of ipRGCs were initially described as relatively insensitive to light, sluggish, and characterized by sustained depolarization and tonic firing rate. The sluggish responses and the ability to maintain stable firing rate under prolonged light exposure makes ipRGCs suitable for long-term irradiance detection. Therefore, we were surprised to observe that *Arvicanthis* ipRGCs showed robust responses to brief light flashes, as shown in Figure 8. Using light stimulation of constant intensity at 14.3 log N_Q_ * s^-1^ * cm^-2^, i.e. a stimulus intensity that produces a distinct response in all three types when presented for 30 s (cf. Figures 6 and 7), type I ipRGCs of *Arvicanthis* also discriminated brief flashes of 10 ms, whereas type II and type III were unresponsive (Figure 8A, B). Type I mouse ipRGCs were very poorly responsive to short flashes, with barely detectable responses to 50 ms, and no responses to 10 ms flashes (Figure 8A, right side). In addition, the response strength at all stimulus intensities for all three classes of mouse ipRGCs was lower than the response of the corresponding type in *Arvicanthis* (compare traces in Figure 8). The mean spike rate and the peak firing rate of type I cells in the *Arvicanthis* retina increased with stimulus duration reaching a maximum at about 1 s, then the rate declined again (Figure 8B, left). Response duration continuously increased with flash duration (Figure 8B, middle), whereas response latency declined to a minimal value and then remained almost constant for stimuli of 50 ms or longer (Figure 8B, right). If light flashes of 10 and 50 ms duration (505 nm; 14.3 log N_Q_ * s^-1^ * cm^-2^) were applied at frequencies between 0.02 Hz and 20 Hz, type I ipRGCs could discriminate light flashes at flicker frequencies up to 0.2 Hz, whereas at higher frequencies the responses merged completely (Figure 9A, B). *Arvicanthis* type II ipRGCs did not respond to single short light flashes, but showed responses to flashes at higher frequency (only merged responses; data not shown). Type III ipRGCs were completely insensitive to short flashes, irrespective of the frequency. In summary, the different sensitivities between the three physiological types of ipRGCs were reflected not only in threshold light intensities, half maximal responses (IR 50) and response parameters for long light stimuli, but also in differential sensitivity to varied pulse lengths.

## Discussion

In this study, we have explored the morphological and physiological properties of ipRGCs in the diurnal rodent *Arvicanthis* *ansorgei* and compared them to data obtained from nocturnal mice. This information was so far lacking for diurnal rodents, and is valuable for assessing the role of retinal inputs in defining temporal niches. The study indicates that the basic physiological and morphological features of ipRGCs in *Arvicanthis* are comparable to mice. However, conspicuous differences were observed with respect to the preponderance of M1 subtype, expression of certain phenotypic markers, firing rate levels and elevated sensitivity of type I *Arvicanthis* ’ ipRGCs to short light flashes. The lack of other clear-cut differences is somewhat surprising when considering that changes in retinal input into the circadian clock within the SCN can apparently mediate temporal niche switching [13].

An animal can confine its activity to a nocturnal or diurnal niche in two ways: 1) through synchronization (entrainment) of an endogenous clock by photic or non-photic timing cues, which then controls activity cycles; and 2) by responding directly to illumination with an activity change (positive or negative masking) [41]. The acute response to light is independent of the circadian clock, but can obscure (mask) the effect of the zeitgeber [42]. Melanopsin-containing ipRGCs are essential for circadian photoentrainment, and are related to masking as well. The role of the retina in determining phasing of activity in diurnal and nocturnal species was demonstrated in mutant mice with melanopsin dysfunction (*Opn4* ^*-/-*^) or in mice lacking melanopsin together with rod function (*Opn4* ^*-/-*^ *; Gnat1* ^*-/-*^), which both can develop diurnal behavior [14,41]. *Rpe65* ^*-/-*^ *; Opn4* ^*-/-*^ mice with targeted deletion of both melanopsin and RPE65 (a protein required for chromophore regeneration in rods and cones), exhibit also a diurnal phenotype, whereas normal photoentrainment is lost [43]. Targeted disruption of *Rpe65* alone, where only part of the rod system remains functional, although with reduced sensitivity [44], leads to a greatly reduced photic input into the circadian system as assessed by phase shifting sensitivity [43], recordings from ipRGCs and estimation of pupillary light response [45]. The switch from nocturnal to diurnal phenotype in *Rpe65* ^*-/-*^ *; Opn4* ^*-/-*^ mice is accompanied by a reversal of clock gene expression in the SCN, together with a change in acute masking effects of light, suggesting determination of temporal niche by neural mechanisms upstream from the SCN [13]. These data may indicate a role of retinal outer photoreception in conjunction with ipRGC function for photoentrainment and for determining diurnal and nocturnal phenotypes. Furthermore, in experiments with wild-type mice where the light intensity during the light-phase of a light: dark cycle was reduced to scotopic levels, these previously nocturnal mice switch to diurnal behavior, emphasizing a strong contribution of rod pathways [13]. Rod signaling mediates circadian photoentrainment in mice through the rod bipolar pathway (at low light intensities), or uses rod signaling through a cone-dependent pathway (at mesopic and photopic conditions) [46]. However, the role of photoreceptor activity in determining nocturnality or diurnality might be overestimated under laboratory conditions, because the activity of mice may be completely diurnal in a natural environment [47,48].

The molecular phenotyping of ipRGCs in *Arvicanthis* (present study) and in mice [20] revealed largely similar expression patterns of RGC markers in diurnal and nocturnal animals, with Brn3 and neurofilaments preferentially expressed in non-M1 ipRGCs as the most crucial feature. However, Brn3, NF and NeuN expression was much lower in *Arvicanthis* ipRGCs compared to mice [20]. Conspicuous differences were seen as the high percentage of M1 cells: ~74% in *Arvicanthis* compared to 30–44% in the mouse retina [20,34,38], and the correspondingly low numbers of the M2 subtype and displaced ipRGCs in *Arvicanthis* . M1 ipRGCs project to brain regions involved in circadian behavior such as the SCN, the intergeniculate leaflet and the ventral lateral geniculate nucleus (LGN), but also to the shell of the olivary pretectal nucleus (OPN) for mediation of the pupillary light reflex [49]. These different innervation patterns possibly originate from molecularly distinct subpopulations of M1 ipRGCs, where Brn3b-negative M1 ipRGCs innervate the SCN and part of the intergeniculate leaflet and Brn3b-positive ipRGCs innervate other targets in the brain including the OPN [50]. The innervation pattern of non-M1 types shares some central targets with the M1 type, including the SCN and OPN [21,49,51], but it also innervates the dorsal LGN and superior colliculus which represent retinotopically organized nuclei mediating object localization and discrimination [52,53]. The heterogeneity of ipRGC central projections might suggest different functions in non-visual photoreception which could also influence temporal niche switching. Anterograde tracing studies in *Arvicanthis* using cholera B toxin showed reduced ipRGC innervation of the ventral subparaventricular zone compared to nocturnal rats [54], although the projection sites of ipRGC subtypes remain unknown.

We observed a 10-fold reduction of ipRGC density in the first 2 postnatal weeks in the *Arvicanthis* retina. A similar, but smaller postnatal loss of ipRGCs has been described in the mouse retina [33,55]. Taking Brn3 as a marker favoring M2 cells, the lower number of Brn3+ cells in P0 retinas compared to adults suggests a lower percentage of M2 cells. Nevertheless, it is difficult to assess if the lower M2 number in newborn retinas results from a delayed development of M2 cells, a delayed melanopsin expression in some M2 cells, or a higher postnatal death in M1 ipRGCs compared to the M2-subtype.

The physiological responses of newborn *Arvicanthis* ipRGCs are generally similar to those in the mouse (this study [33]). Three different cell types (I, II and III) characterized by their response pattern and sensitivities could be clearly distinguished in both animals with comparable response parameters, despite the elevated firing rate of *Arvicanthis* ipRGCs and higher sensitivity of *Arvicanthis* type I ipRGCs to short light flashes. IpRGCs were originally described as sluggishly responding cells with slow response kinetics [8], so it was unexpected to observe pronounced responses to light flashes as short as 10 ms. This observation is supported by the described ability of ipRGCs to signal single-photon absorption [56]. Only type I ipRGC responded to short flashes (10 and 50 ms), adding a new feature to their description as the most sensitive ipRGC type. Moreover, it indicates an extremely large range of ipRGCs’ sensitivity, showing responses to millisecond flashes up to several hours of illumination [57]. We were unable to definitively attribute a given physiological type to a distinct morphological type (M1 or M2), particularly in newborn retinas in which classification is difficult. We assume that our physiologically characterized type I correlate with the type III of Tu et al. [33] showing the highest sensitivity, shorter response latency and longer response duration than those of the other types. This cell population possibly corresponds to brightly melanopsin-positive M1 ipRGCs. The M1 identity is supported by recordings from morphologically identified ipRGCs demonstrating a higher sensitivity compared to other types [37]. This M1-subtype comprises the major input to the SCN [21]. The other two physiologically identified ipRGC types in *Arvicanthis* have their morphological correlate in the non-M1 (M2) cell population. The most numerous type II in *Arvicanthis* (59%) corresponds to the type I of Tu et al. [33], which comprises about 72% of the ipRGCs in the mouse retina, and type III in *Arvicanthis* correlates with type II in the mouse.

The similar morphological and physiological properties of ipRGCs of the nocturnal and diurnal species raise questions about the functional significance of the non-visual system in determining the temporal niche. In addition to ipRGCs, retinal rods and cones provide light information for photo-entrainment [10,12,58]. In ipRGCs from adult retinas, light stimulation evokes intrinsic, melanopsin-based photoresponses as well as extrinsic, synaptically-driven responses [59]. Rods signal light to M1 ipRGCs either directly via bipolar axonal synapses [60], or indirectly via the rod bipolar to AII amacrine cell pathway [61]. Cones use primarily the ON pathway as dominant excitatory input to ipRGCs at bright light intensities what affects mainly the light responses in M2 ipRGCs, and only to a minor extent the M1 cells suggesting that non-image forming vision is relayed through different ipRGC subtypes to various brain centers [62]. If temporal niche switching is determined or modulated by channeling light through distinct rod- or cone-based pathways to ipRGCs, as suggested by Doyle et al. [13], these mechanisms might influence diurnality or nocturnality in an otherwise similar non-image forming visual system. The large cone contingent (~30% of all photoreceptors) in *Arvicanthis* [18], compared to ~3% in mice [63], might play an influential role in this respect. Indeed, specific adaptations to a nighttime or daytime niche are commonly seen in the retina, with well-developed vision and high cone density in diurnal animals, and rod-dominated retinas in nocturnal animals (for review see 48).

In conclusion, the higher light sensitivity and elevated proportion of M1 cells in *Arvicanthis* might, together with their cone-rich retina, change the coding of ambient illumination and play a role in shaping their circadian behavior.
